# Retraction: Golgi integral membrane protein 4 manipulates cellular proliferation, apoptosis and cell cycle in human head and neck cancer

**DOI:** 10.1042/BSR20180454_RET

**Published:** 2026-05-21

**Authors:** Xiaobo Cui, Yunfei Bai, Dongxue Gao, Yaping Wang, Boqian Wang, Wei Wang

**Affiliations:** Inner Mongolia Medical University Affiliated Hospital, Hohhot, Inner Mongolia, People's Republic of China

**Keywords:** apoptosis, cell cycle, GOLIM4, head and neck cancer, proliferation

This article is being retracted from *Bioscience Reports* at the request of the Editor-in-Chief and the Editorial Board. This follows the receipt of a notification from a reader, alerting the Editorial Board to a similarity between this manuscript and a paper published in another journal by different authors. Upon investigation, the Editorial Office identified two further similarities. Specifically, these involve the following data:

- The Figure 2D GOLIM4 bands have high similarity to the Figure 6C AKT bands from Gong et al. 2015 (DOI: 10.18632/oncotarget.4624).
- The Figure 2D GOLIM4 bands also seem to appear in the Fig 2D SNRPA1 bands from Feng et al. 2020 (DOI: 10.1042/BSR20193815 [retracted])
- The Figure 2D GAPDH bands also appear as the Fig 6D GAPDH bands from Wang et al. 2018 (DOI: 10.1042/BSR20180574 [retracted])

The authors have been contacted with regards to the concerns and provided raw data; however, concerns remain surrounding the raw data provided. Given the extent of the issues raised, the Editorial Board stands by the decision to retract the article. The authors disagree to the retraction.

